# Supplementary material for: Phosphatidylserine exposure mediated by ABC transporter activates the integrin signaling pathway promoting axon regeneration
Source: Nat Commun. 2018 Aug 6;9:3099. doi: 10.1038/s41467-018-05478-w (PMC6079064; doi:10.1038/s41467-018-05478-w)
Supplement: Supplementary file 1 — Supplementary information [file 41467_2018_5478_MOESM1_ESM.pdf]

Phosphatidylserine exposure mediated by ABC transporter activates the integrin signaling pathway promoting axon regeneration

Hisamoto *et al*.

Supplementary Tables and Figures

Supplementary Table 1. Raw data for genotypes tested by axotomy.

Strain	Genotype *(juIs76* background)	Regeneration	Total	% of regeneration	*P* value
KU501	wild type	39	52	75	-
KU747	*ttr-57(km6877)*	41	53	77	0.82[a]
KU64	*ttr-11(km64)*	26	53	49	0.009[a]
KU748	*ttr-11(km64); Ex[P_{ttr-11}::ttr-11]*	39	53	74	0.016[b]
KU750	*ttr-11(km64); Ex[P_{mec-7}::ttr-11]*	38	50	76	0.008[b]
KU749	*ttr-11(km64); Ex[P_{ttr-11}::ttr-11(N46A)]*	28	54	52	0.85[b]
KU723	*ina-1(gm39)*	30	61	49	0.007[a], [†]
KU711	*ced-10(n3246)*	21	51	41	<0.001[a], [†]
KU751	*ttr-11(km64); ina-1(gm39)*	25	50	50	1[b]
KU752	*ttr-11(km64); ced-10(n3246)*	22	50	44	0.69[b]
KU753	*ttr-11(km64); Ex[P_{unc-25}::ced-10(G12V)]*	39	51	76	0.005[b]
KU754	*ttr-11(km64); Ex[P_{unc-25}::ced-10(T17N)]*	21	52	40	0.43[b]
KU755	*anoh-1(tm4762)*	35	52	67	0.52[a]
KU756	*ced-8(n1891)*	34	53	64	0.29[a]
KU776	*anoh-1(tm4762); ced-8(n1891)*	35	54	65	0.29[a]
KU757	*ced-7(n2094)*	26	55	47	0.005[a]
KU758	*chat-1(ok1681)*	44	51	86	0.21[a]
KU759	*ced-7(n2094); chat-1(ok1681)*	42	55	76	0.003[c]
KU760	*ced-7(n2094); ttr-11(km64)*	24	52	46	1[c]
KU777	*ced-7(n2094); Ex[P_{unc-25}::ced-10(G12V)]*	41	53	77	0.001[c]
KU761	*ced-7(n2094); Ex[P_{unc-25}::ced-7]*	39	50	78	0.001[c]
KU762	*ced-7(n2094); Ex[P_{unc-25}::ced-7(D1691A)]*	27	60	45	0.85[c]
KU763	*ced-3(ok2734)*	22	54	41	<0.001[a]
KU764	*ced-3(ok2734); ced-7(n2094)*	34	72	47	0.59[d]
KU765	*ced-3(ok2734); Ex[P_{unc-25}::ced-7]*	29	58	50	0.35[d]
KU766	*ced-3(ok2734); Ex[P_{unc-25}::ced-7ΔC]*	38	59	64	0.01[d]
KU767	*crt-1(jh101)*	22	50	44	0.002[a]
KU64*	*ttr-11(km64)*	24	52	46	-
KU85*	*ttr-11(km64) ttr-57(km85)*	30	54	56	0.44[e]
KU785*	*ttr-11(km64) ; Ex[P_{ttr-57}::ttr-57]*	24	50	48	1[e]
KU784*	*ttr-11(km64) ; Ex[P_{unc-25}::ttr-11]*	24	51	47	1[e]
KU757*	*ced-7(n2094)*	21	50	42	-
KU778*	*ced-7(n2094); Ex[P_{ttr-11}::ttr-11]*	42	64	66	0.014[f]
KU779*	*ced-7(n2094); Ex[P_{mec-7}::ced-7],* cut D only	14	38	37	0.67[f]
KU779*	*ced-7(n2094); Ex[P_{mec-7}::ced-7],* cut D + PLM	27	38	71	0.005[f]

Strain	Genotype *(wpIs36* background)	Regeneration	Total	% of regeneration	*P* value
KU769	wild type	26	50	52	-
KU770	*Ex[P_{hsp}::ss::mfg-e8-c2::gfp]*	12	54	22	0.002[g]
KU771	*Ex[P_{hsp}::ss::mfg-e8-c2(AAA)::gfp]*	25	53	47	0.695[g]

a: vs wild type, b: vs KU64, c: vs KU757, d: vs KU763, e: vs KU64*, f: vs KU757*, g: vs KU769

*: Supplementary Figures 2, 10 and 11b.

[†]: Data from previous results[11]

Supplementary Table 2. Strains used in this study.

KU501	*juIs76 II*
KU64	*juIs76 II; ttr-11(km64) V*
KU85	*juIs76 II; ttr-11(km64) ttr-57(km85) V*
KU711	*juIs76 II; ced-10(n3246) IV*
KU723	*ina-1(gm39)/hT2[bli-4(e937) let-(q782) qIs48] (I;III); juIs76 II*
KU747	*juIs76 II; ttr-57 (tm6877) V*
KU748	*juIs76 II; ttr-11(km64) V; kmEx748 [P_{ttr-11}::ttr-11]*
KU749	*juIs76 II; ttr-11(km64) V; kmEx749 [P_{ttr-11}::ttr-11(N46A)]*
KU750	*juIs76 II; ttr-11(km64) V; kmEx750 [P_{mec-7}::ttr-11]*
KU751	*ina-1(gm39)/hT2[bli-4(e937) let-(q782) qIs48] (I;III); juIs76 II; ttr-11(km64)V*
KU752	*juIs76 II; ced-10(n3246) IV; ttr-11(km64)V*
KU753	*juIs76 II; ttr-11(km64) V; kmEx466 [P_{unc-25}::ced-10(G12V)]*
KU754	*juIs76 II; ttr-11(km64) V; kmEx467 [P_{unc-25}::ced-10(T17N)]*
KU755	*juIs76 II; anoh-1(tm4762) III*
KU756	*juIs76 II; ced-8 (n1891) X*
KU757	*juIs76 II; ced-7(n2094) III*
KU758	*juIs76 II; chat-1(ok1681)/ nT1[qIs51] (IV;V)*
KU759	*juIs76 II; ced-7(n2094) III; chat-1(ok1681)/ nT1[qIs51] (IV;V)*
KU760	*juIs76 II; ced-7(n2094) III; ttr-11(km64)V*
KU761	*juIs76 II; ced-7(n2094) III; kmEx761 [P_{unc-25}::ced-7]*
KU762	*juIs76 II; ced-7(n2094) III; kmEx762 [P_{unc-25}::ced-7(D1691A)]*
KU763	*juIs76 II; ced-3(ok2734) IV*
KU764	*juIs76 II; ced-7(n2094) III; ced-3(ok2734) IV*
KU765	*juIs76 II; ced-3(ok2734) IV; kmEx761 [P_{unc-25}::ced-7]*
KU766	*juIs76 II; ced-3(ok2734) IV; kmEx766 [P_{unc-25}::ced-7ΔC]*
KU767	*juIs76 II; crt-1(jh101) V*
KU769	*wpIs36 I*
KU770	*wpIs36 I; kmEx770 [P_{hsp}::ss::mfg-e8-c2::gfp]*
KU771	*wpIs36 I; kmEx771 [P_{hsp}::ss::mfg-e8-c2(AAA)::gfp]*
KU772	*wpIs36 I; ced-7(n2094) III; kmEx770 [P_{hsp}::ss::mfg-e8-c2::gfp]*
KU773	*wpIs36 I; ced-3(ok2734) IV; kmEx770 [P_{hsp}::ss::mfg-e8-c2::gfp]*
KU774	*wpIs36 I; crt-1(jh101) V; kmEx770 [P_{hsp}::ss::mfg-e8-c2::gfp]*
KU775	*wpIs36 I; ttr-11(km64) V; kmEx770 [P_{hsp}::ss::mfg-e8-c2::gfp]*
KU776	*juIs76 II; anoh-1(tm4762) III; ced-8 (n1891) X*
KU777	*juIs76 II; ced-7(n2094) III; kmEx466 [P_{unc-25}::ced-10(G12V)]*
KU778	*juIs76 II; ced-7(n2094) III; kmEx748 [P_{ttr-11}::ttr-11]*
KU779	*juIs76 II; ced-7(n2094) III; kmEx779 [P_{mec-7}::ced-7]*
KU780	*kmIs10; kmEx780 [P_{ttr-11}::nls::venus]*
KU781	*kmIs10; kmEx781 [P_{ttr-11}::ttr-11::gfp]*
KU782	*wpIs36 I; kmEx782 [P_{unc-25}::ss::mfg-e8-c2::gfp]*
KU783	*wpIs36 I; kmEx783 [P_{hsp}::ss::anxv::gfp]*
KU784	*juIs76 II; ttr-11(km64) V; kmEx784 [P_{unc-25}::ttr-11]*
KU785	*juIs76 II; ttr-11(km64) V; kmEx785 [P_{ttr-57}::ttr-57]*

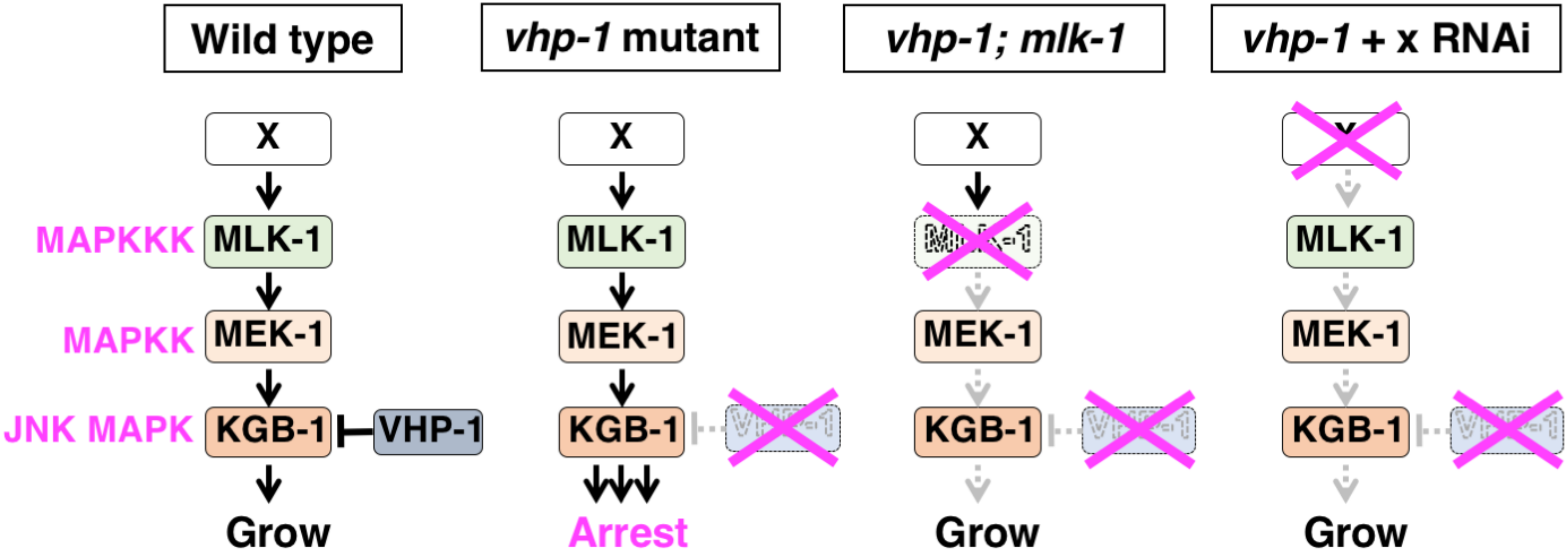

Supplementary Figure 1. Isolation of *svh*. In *vhp-1* mutants, hyperactivation of the KGB-1 JNK cascade causes larval arrest. Downregulation by RNAi of any of the components of the JNK pathway, for example *mlk-1*, or a factor that positively regulates the activity of the KGB-1 signaling is sufficient to suppress mutant *vhp-1* lethality.

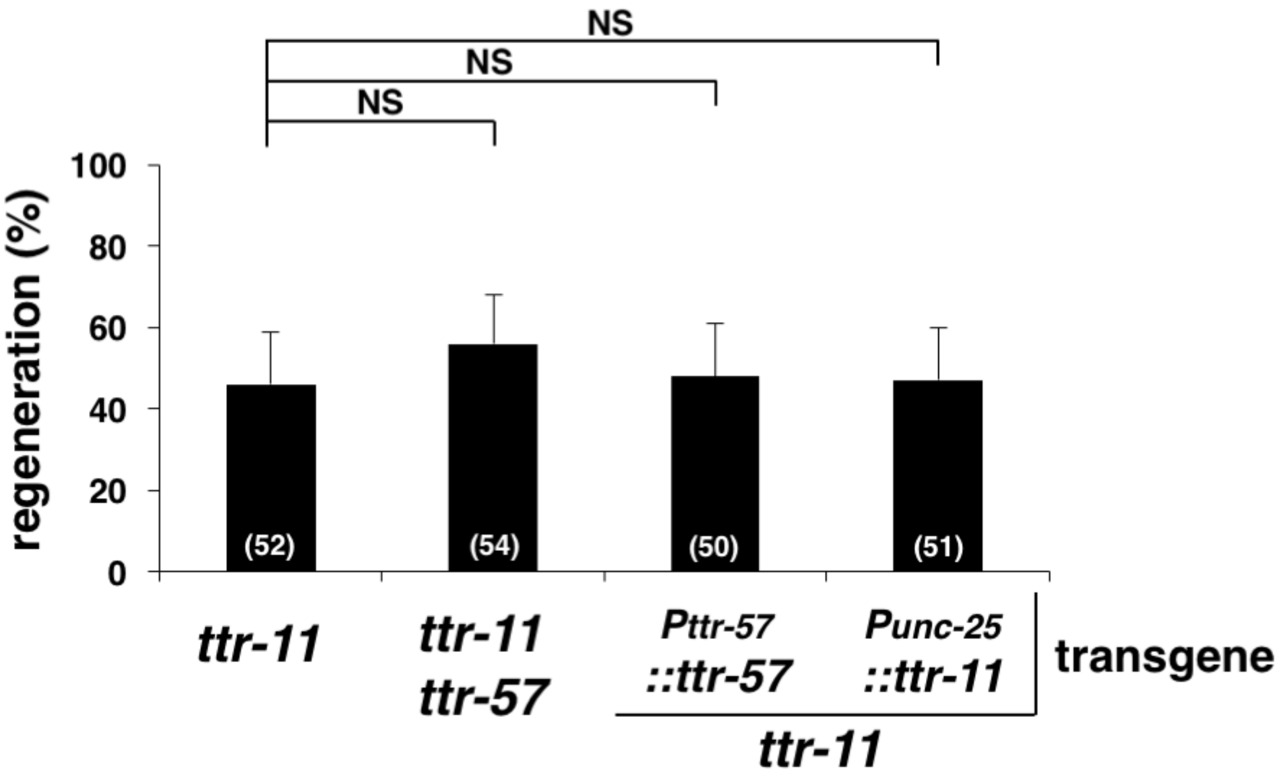

Supplementary Figure 2. Effects of *ttr-11* and *ttr-57* on axonal regeneration. Percentages of axons that initiated regeneration 24 hr after laser surgery in the L4 stage are shown. The numbers (n) of axons examined are shown. Error bars indicate 95% confidence intervals (CI). NS: not significant as determined by Fisher's exact test.

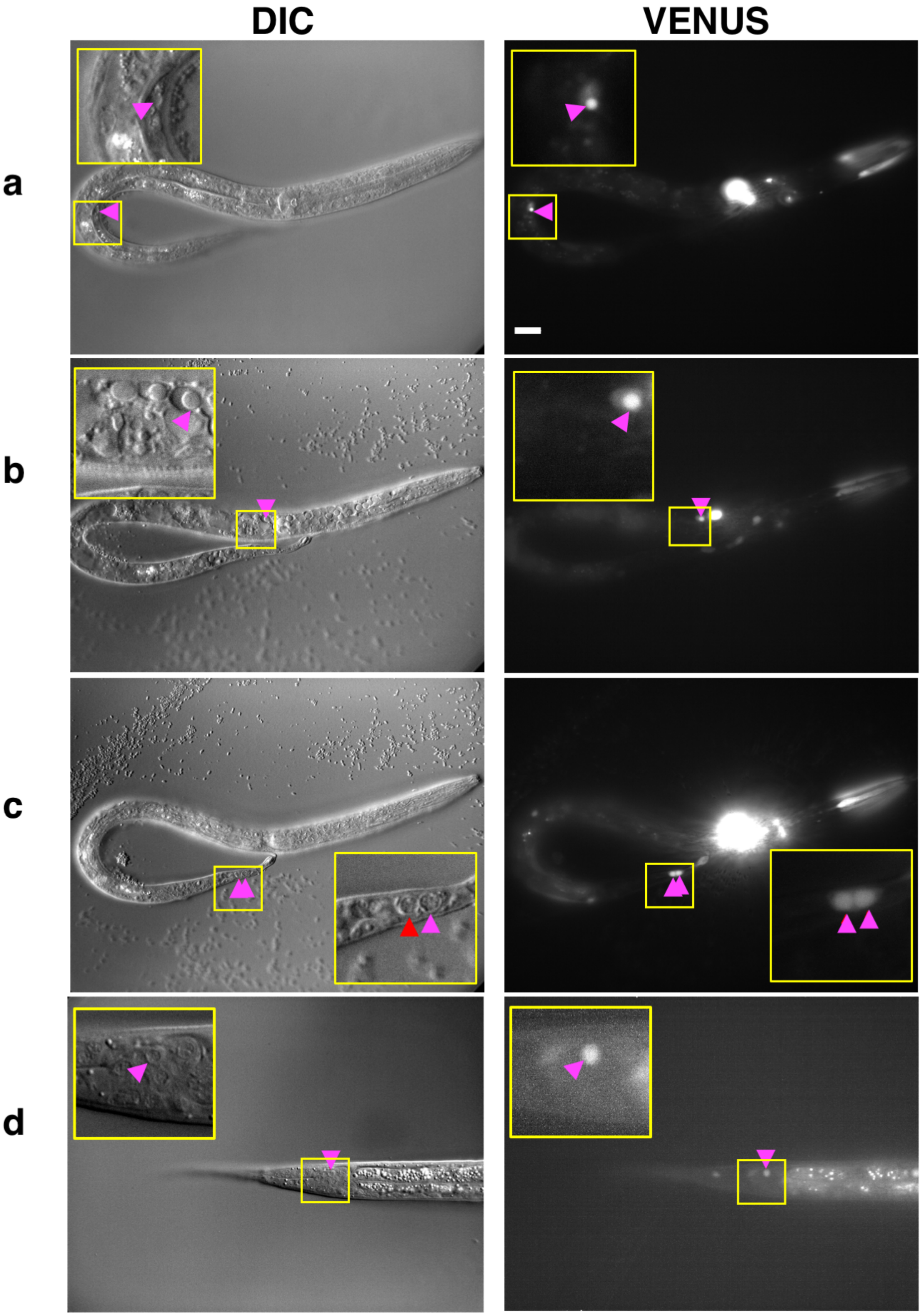

Supplementary Figure 3. Expression of the *Pttr-11::nls::venus* gene. DIC and fluorescent images of L1 animals carrying the *Pttr-11::nls::venus* and *Punc-25::nes ::cfp* transgenes are shown. The signal in the head is from the injection marker *Pttx-3::gfp*. Magenta arrowheads indicate cells expressing NLS-VENUS (**a** HSN neuron; **b** excretory gland cell; **c** hyp10 cells; **d** DVA neuron). The regions in the yellow squares are shown magnified in the insets. Ten animals were examined and all showed a similar expression pattern. Scale bar = 10 µm.

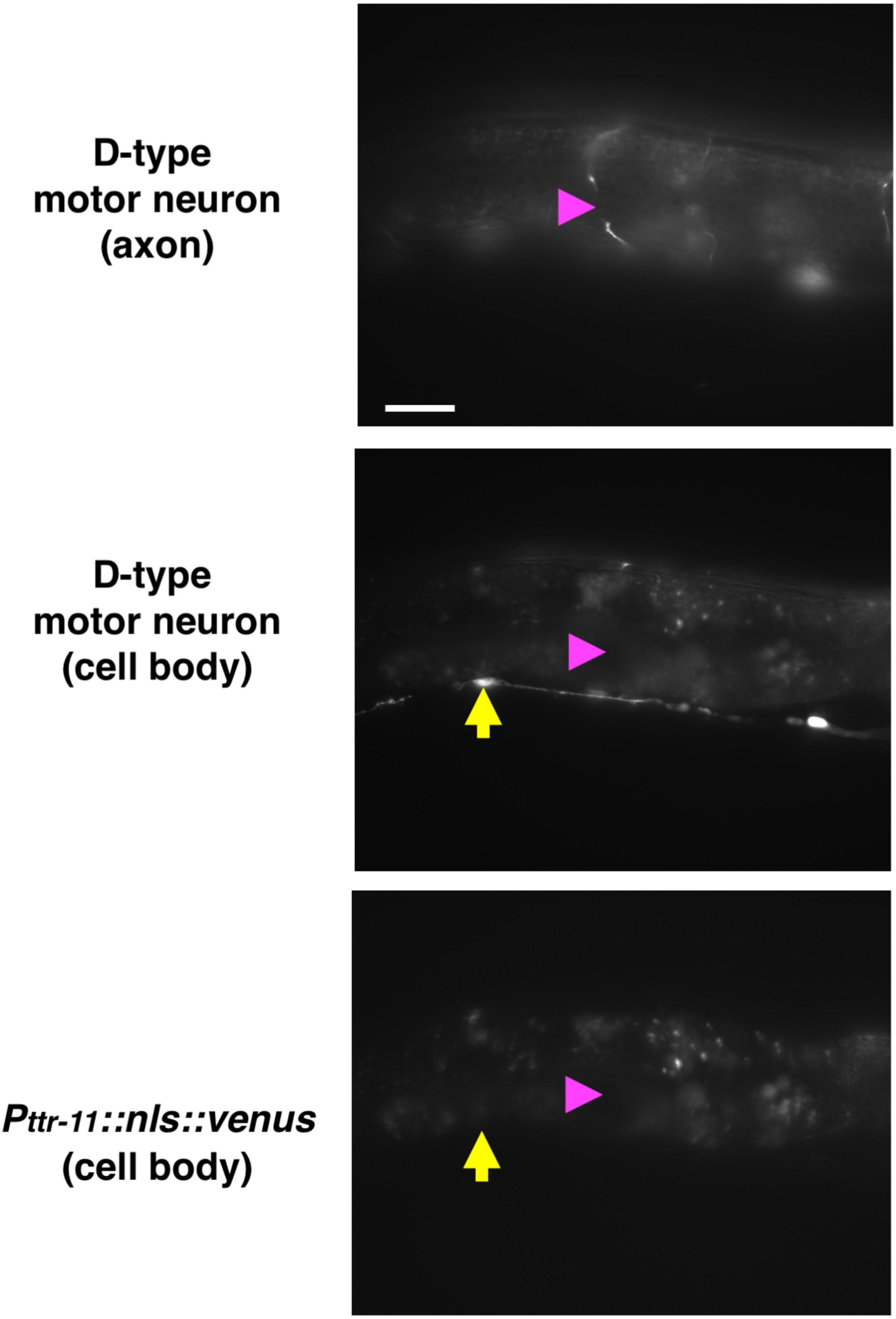

Supplementary Figure 4. Expression of *Pttr-11::nls::venus* after axon injury. Fluorescent images of L4 animals carrying the *Pttr-11::nls::venus* and *Punc-25::nes ::cfp* transgenes are shown. Images were taken at 30 min after laser surgery. D neurons are visualized by CFP under control of the *unc-25* promoter. Magenta arrowheads and yellow arrows indicate the site of axotomy and the cell body of the injured neuron, respectively. Five animals were examined and all showed a similar pattern of no detectable *Pttr-11::nls::venus* expression in the injured neuron. Scale bar = 10 μm.

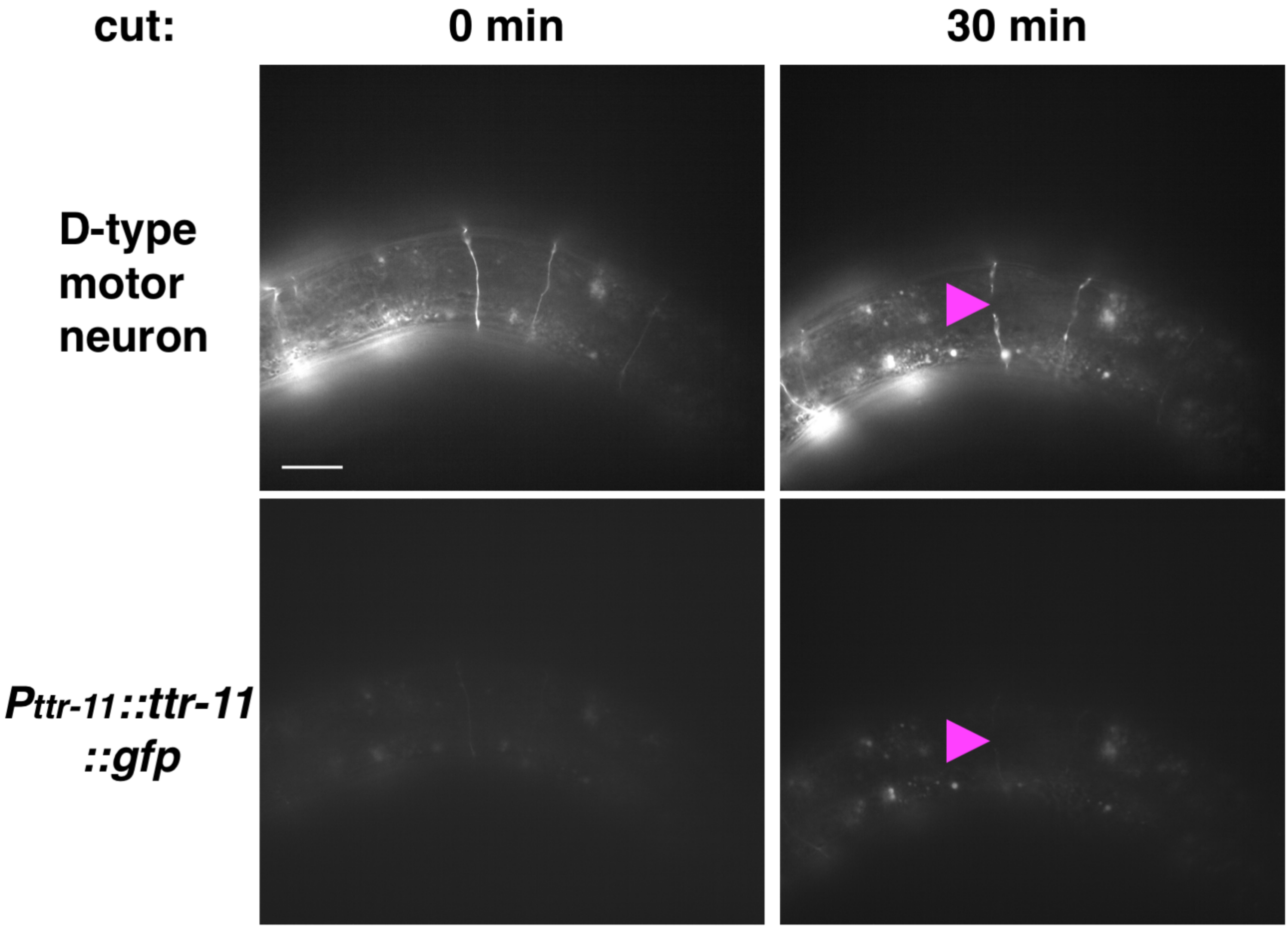

Supplementary Figure 5. Expression of *Pttr-11::ttr-11::gfp* after axon injury. Fluorescent images of L4 animals carrying the *Pttr-11::ttr-11::gfp* and *Punc-25::nes::cfp* transgenes are shown. Images were taken at 0 and 30 min after laser surgery. D neurons are visualized by CFP under control of the *unc-25* promoter. Magenta arrowheads indicate the site the site of axotomy. Apart from tissue autofluorescence, no signal from the *Pttr-11::ttr-11::gfp* transgene was detected. Twenty animals were examined and all showed the same pattern. Scale bar = 10 µm.

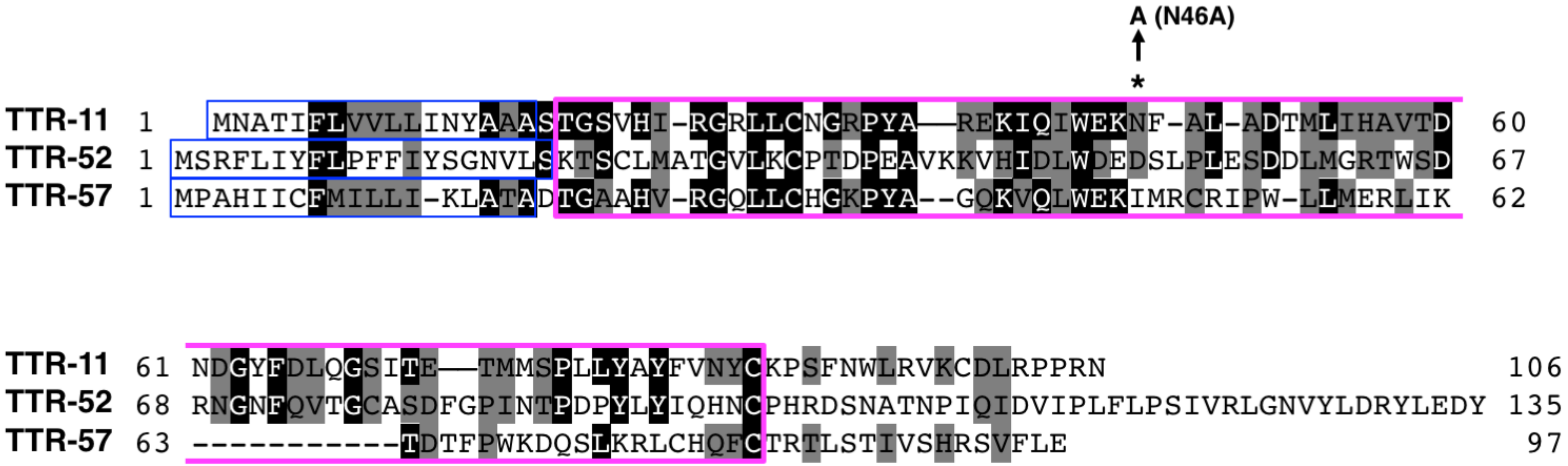

Supplementary Figure 6. Sequence alignments of TTR-11,TTR-52 and TTR-57. Identical residues are shaded in black, and similar residues in grey. A residue essential for the function is marked with an asterisk. Blue boxes indicate the predicted secretion signal. Magenta box delineates the transthyretin-like domain.

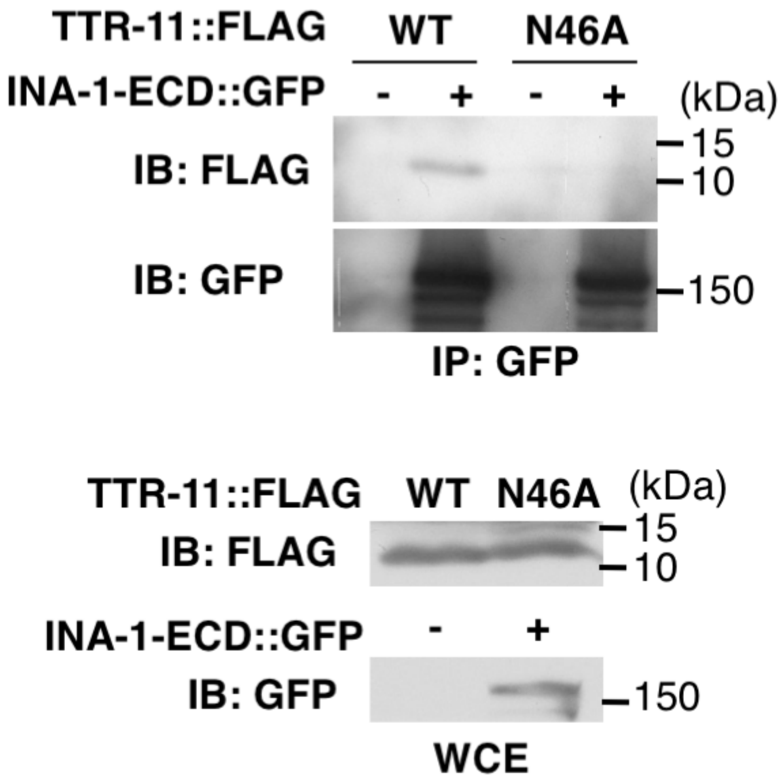

Supplementary Figure 7. Interaction of TTR-11 with the INA-1 extracellular domain in vitro. HEK293 cells were transfected with plasmids encoding INA-1-ECD::GFP and TTR-11::FLAG (wild type and N46A) separately. Cell extracts were prepared from each cell and mixed in vitro. Complex formation was detected by immunoprecipitation (IP) with an anti-GFP antibody, followed by immunoblotting (IB) with an anti-FLAG antibody. Whole-cell extracts (WCE) were analyzed by immunoblotting.

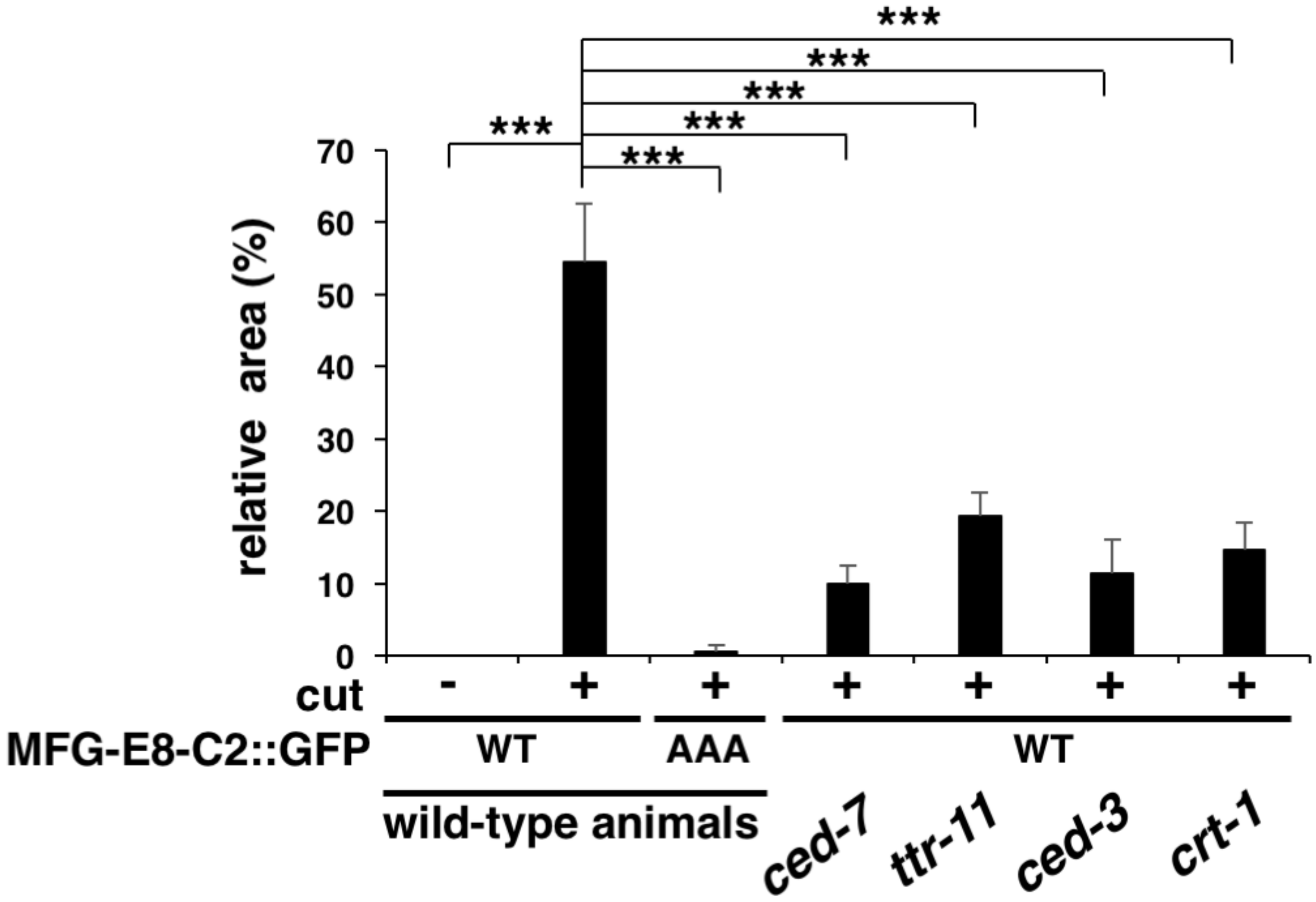

Supplementary Figure 8. Quantification of MFG-E8-C2::GFP. Percentages of relative area around the injury site exhibiting at least a two-fold increase in MFG-E8-C2::GFP fluorescence are shown. Scores were taken at 1 hr after laser surgery. Quantification of MFG-E8-C2::GFP is described in Methods. Twenty animals were examined for each condition. Error bars indicate SEM. ***P<0.001 as determined by unpaired *t*-test.

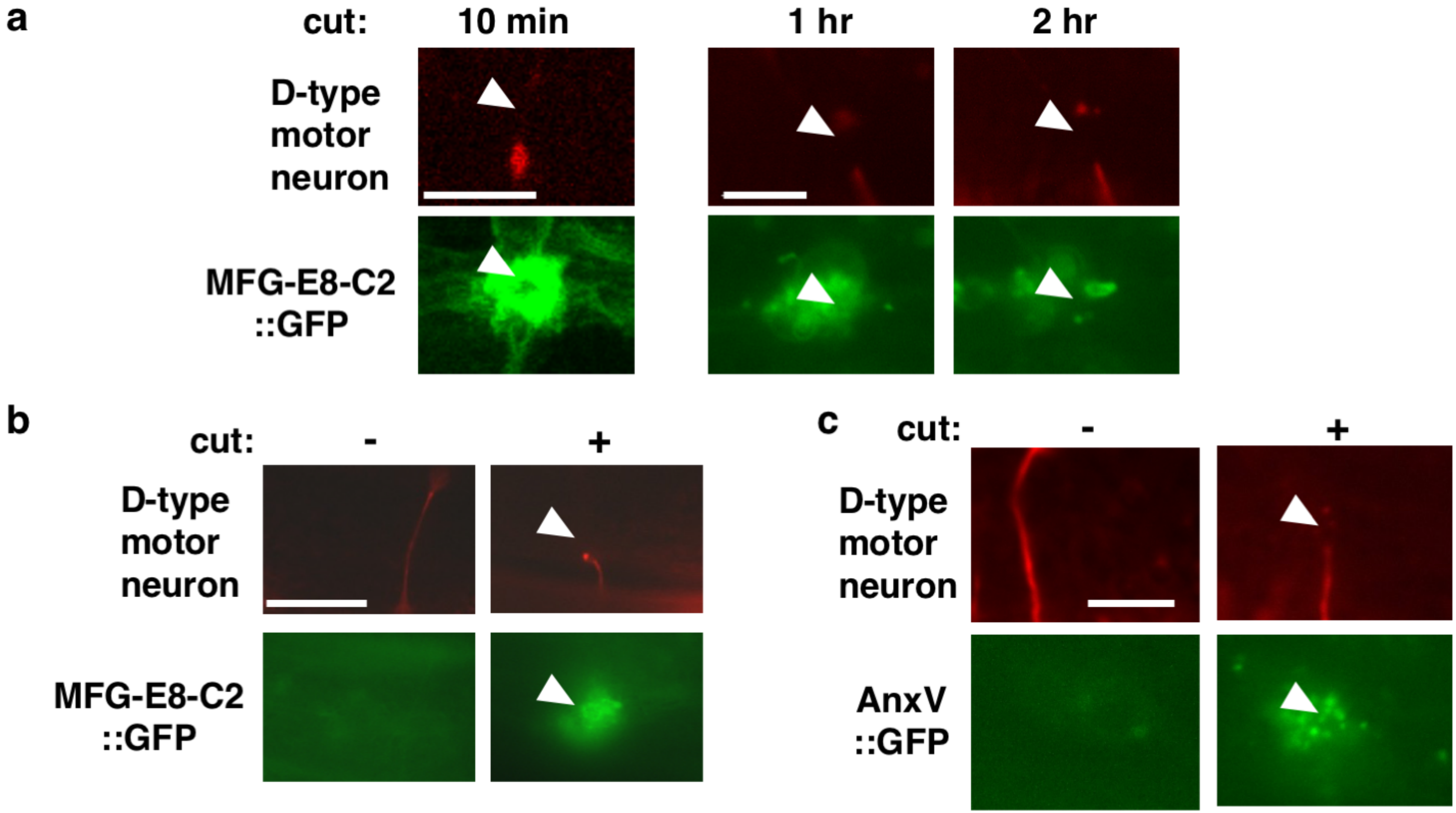

Supplementary Figure 9. Localization of PS after axon injury. **a** Fluorescent images of severed axons in animals carrying *Phsp::ss::mfg-e8-c2::gfp* and *Punc-47::mcherry*. D neurons are visualized by mCherry under control of the *unc-47* promoter. Images were taken at indicated times after laser surgery. Five animals were examined and they showed a similar pattern. Arrowheads indicate the sites of laser surgery. **b** Fluorescent images of severed axons in animals carrying *Punc-25::ss::mfg-e8-c2::gfp* and *Punc-47::mcherry*. Images were taken with (+; 1 hr after laser surgery) or without (-) laser surgery. Twenty animals were examined and showed the same pattern of fluorescence. Arrowheads indicate the sites of laser surgery. **c** Fluorescent images of severed axons in animals carrying *Phsp::ss::anxv::gfp* and *Punc-47::mcherry*. Images were taken with (+; 1 hr after laser surgery) or without (-) laser surgery. Twelve animals expressing moderate amounts of AnxV::GFP showed a similar pattern. Arrowheads indicate the sites of laser surgery. Scale bars = 5 μm.

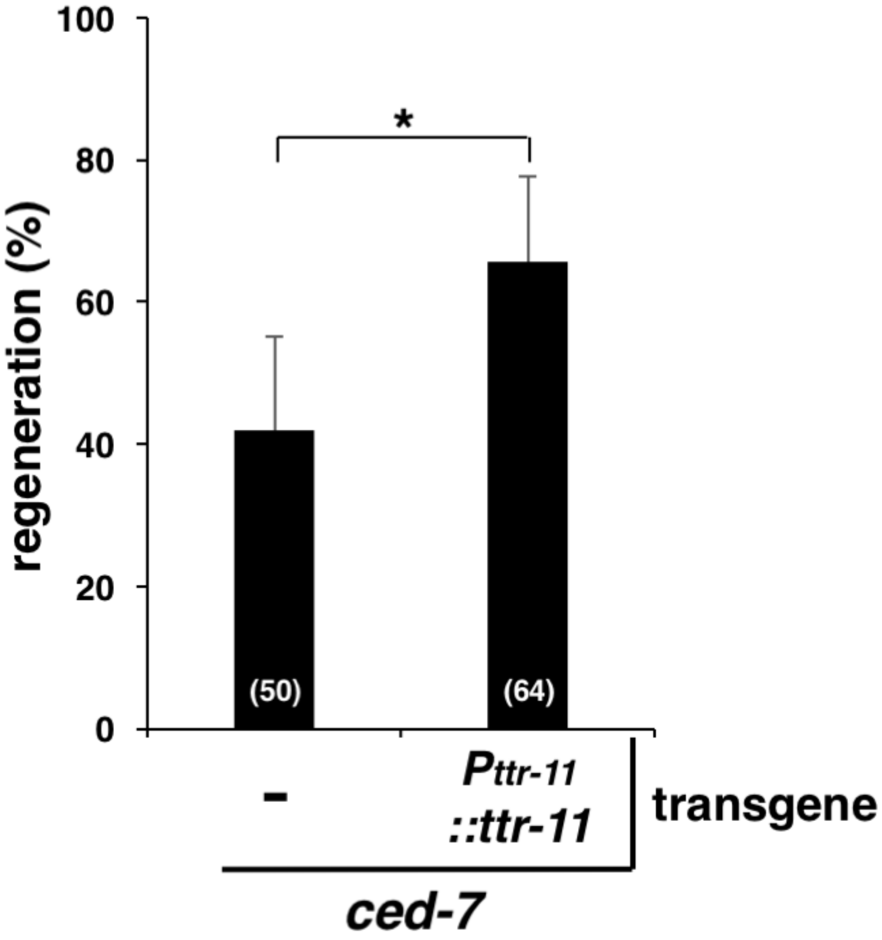

Supplementary Figure 10. The effect of *ttr-11* overexpression on the *ced-7* defect in axon regeneration. Percentages of axons that initiated regeneration 24 hr after laser surgery in the L4 stage are shown. The numbers (n) of axons examined are shown. Error bars indicate 95% CI. *P<0.05 as determined by Fisher's exact test.

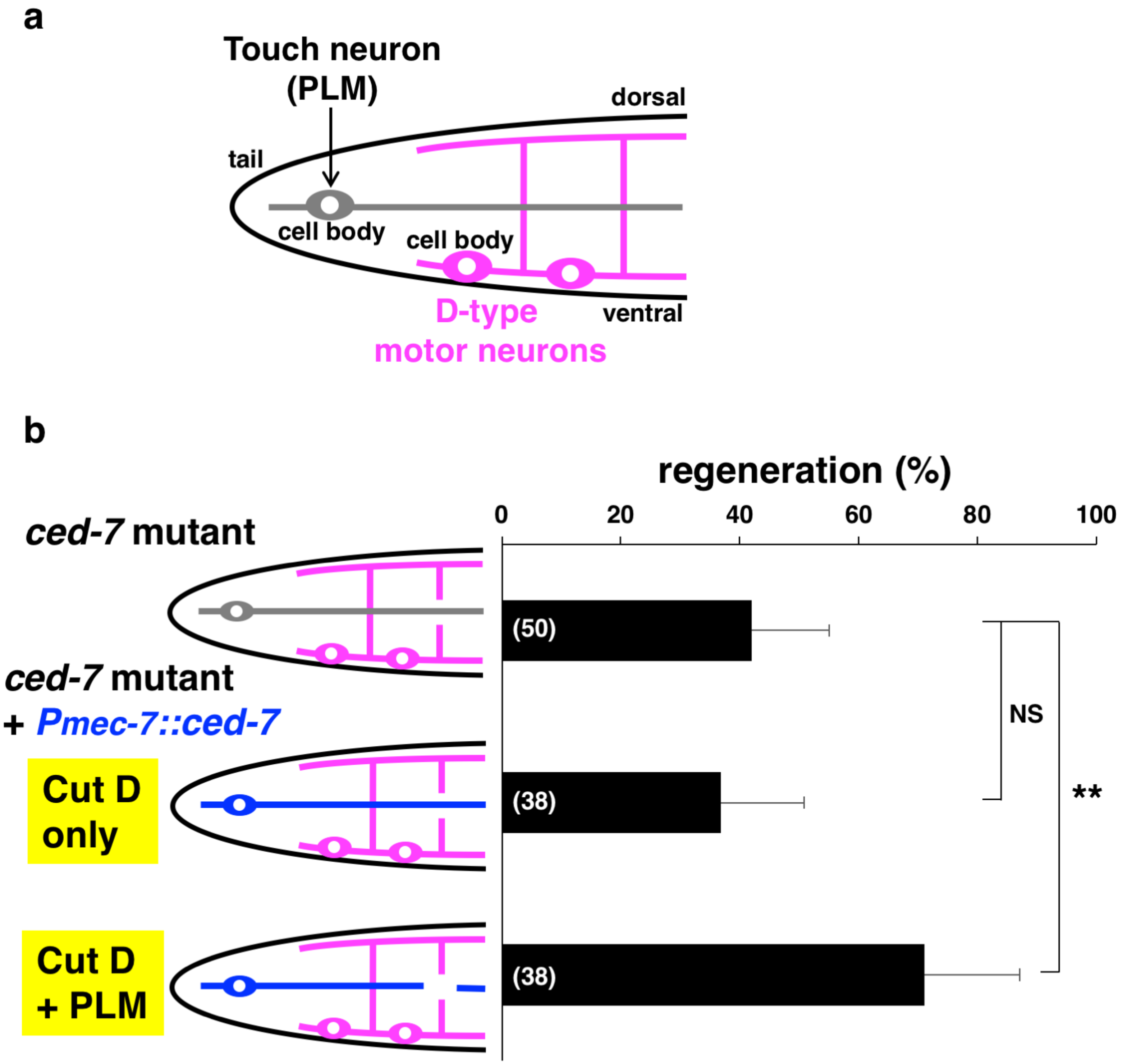

Supplementary Figure 11. The effect of *ced-7* expression in touch neuron on regeneration of D-type motor axon. **a** Schematic drawing of motor and touch neurons. The D-type motor neurons (magenta) have cell bodies on the ventral side and extend axonal commissures dorsally. The touch neuron (gray) extends a long axon parallel to the long body axis and crosses almost perpendicular to the axons of the D-type motor neurons. **b** Percentages of D-type motor axons that initiated regeneration 24 hr after laser surgery in the L4 stage. Schematic drawings of motor and touch neurons are shown in the left part. Blue indicates touch neuron of *ced-7* mutants expressing the *ced-7* gene. VD9, DD5 and VD10 neurons were cut. The numbers (n) of axons examined are shown. Error bars indicate 95% CI . $^{**}P<0.01$ as determined by Fisher's exact test. NS: not significant.

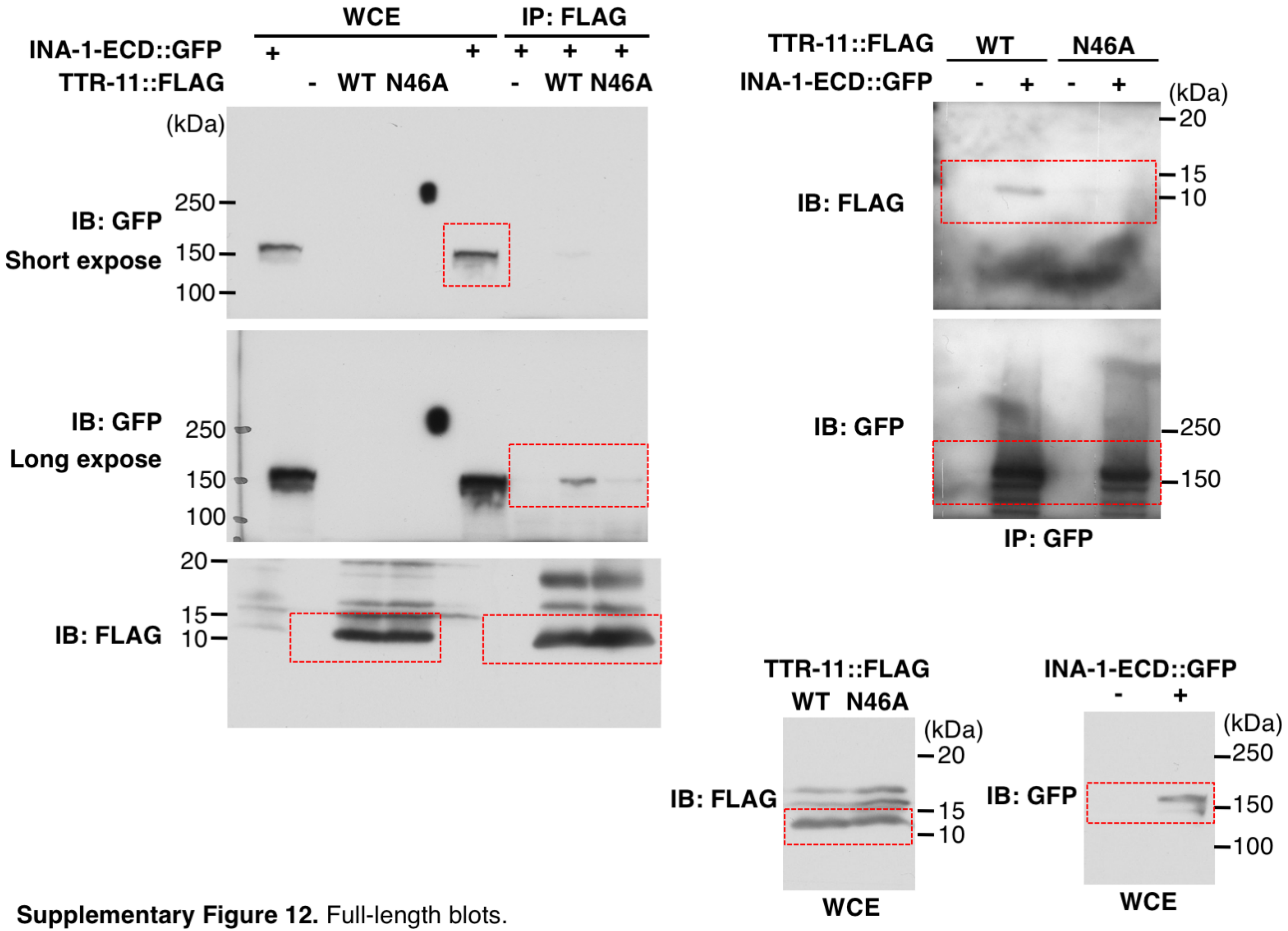

Supplementary Figure 12. Full-length blots.
